# Phenotypic discordance in semilobar holoprosencephaly: a case report of total arhinia and median clefting linked to gestational diabetes

**DOI:** 10.1097/MS9.0000000000005512

**Published:** 2026-08-19

**Authors:** Janu Chhetri, Rejina Chhetri

**Affiliations:** a Department of Diagnostic Radiology and Imaging, Fatima Jinnah Medical University, Lahore, Pakistan; b Department of Pediatric Surgery, Nepalgunj Medical College, Nepalgunj, Nepal

**Keywords:** arhinia, case report, gestational diabetes, neonatal seizures, semilobar holoprosencephaly, Sonic hedgehog signaling

## Abstract

**Introduction:**

Holoprosencephaly (HPE) is a complex spectrum of cephalic malformations resulting from incomplete prosencephalic division. Semilobar HPE is an intermediate variant, often associated with significant craniofacial dysmorphism and neurological morbidity.

**Case presentation:**

We present a preterm female infant born to a mother with unmanaged gestational diabetes. At birth, she exhibited microcephaly, total arhinia, and a median cleft lip and palate. MRI confirmed semilobar HPE. Clinical progression included hypotonia, seizures, and recurrent hyperpyrexia associated with hypothalamic dysfunction. Despite the absence of a nasal airway, the infant transitioned to reflexive mouth breathing and required long-term orogastric tube feeding. Notably, she has maintained systemic stability throughout an 8-month follow-up, facilitated by a proactive, multidisciplinary home-based care model.

**Discussion:**

This case illustrates a phenotypic discordance in which severe arhinia occurs alongside intermediate cerebral cleavage, suggesting a potential vulnerability in which nasal placode development may be highly sensitive to maternal hyperglycemia, which is hypothesized to perturb cholesterol-dependent Sonic Hedgehog signaling. Our clinical experience demonstrates that by prioritizing rigorous caloric titration and proactive monitoring of autonomic and infectious triggers, it is possible to successfully navigate the patient’s precarious equilibrium and prevent the secondary morbidity typically associated with hypothalamic instability.

**Conclusion:**

This report demonstrates that even in the presence of profound structural and autonomic vulnerabilities, proactive multidisciplinary management can effectively mitigate secondary systemic morbidity and support survival beyond the neonatal period. This approach provides a viable model for stabilizing patients with severe holoprosencephaly through consistent, home-based surveillance and functional support.

## Introduction

Holoprosencephaly (HPE) represents a fundamental structural failure of ventral forebrain induction, resulting in incomplete prosencephalic cleavage during the 18th–28th days of gestation^[^1,2^]^. While the intrauterine prevalence is estimated to be as high as 1 in 250 conceptuses, the majority of affected embryos are lost to spontaneous abortion, leading to a live-birth incidence typically estimated to be between 1 in 8000 and 1 in 16 000^[^2^]^. The semilobar variant represents an intermediate architectural phenotype in which posterior hemispheric separation initiates, but anterior structures remain fused. This neuroanatomical derangement is frequently mirrored by a severe constellation of midline craniofacial anomalies, traditionally summarized by the clinical axiom that “the face predicts the brain”^[^3,4^]^.

HIGHLIGHTS

- The case reports rare semilobar holoprosencephaly presenting with total arhinia.
- Neuroimaging confirmed a phenotypic discordance between facial and brain structures.
- Gestational diabetes is highlighted as a plausible environmental metabolic trigger.
- An 8-month survival was achieved via home-based palliative care.
- Proactive multidisciplinary management successfully prevented secondary morbidity.

Among these anomalies, congenital arhinia, the total structural absence of the external nose and internal nasal passages, is an exceptionally rare midline manifestation, signifying a profound disruption of early nasal placode development^[^5^]^. The etiopathogenesis of HPE is fundamentally multifactorial, arising from complex genetic mutations within the Sonic Hedgehog (SHH), ZIC2, and SIX3 pathways, interacting with maternal environmental modulators such as poorly controlled gestational diabetes^[^2,6^]^. In resource-limited settings where definitive molecular testing is unavailable, meticulous clinical evaluation paired with high-resolution postnatal neuroimaging remains the cornerstone of diagnostic mapping and structural risk stratification.

We present a rare case of a female infant with complete congenital arhinia, median cleft lip and palate, and semilobar HPE, born to a mother with unmanaged gestational diabetes. By documenting her structural anatomy, the diagnostic challenges encountered in a resource-limited environment, and her subsequent 8-month longitudinal survival characterized by a persistent reliance on orogastric (OG) tube feeding, this report highlights the non-linear nature of midline embryogenesis and the potential for long-term multidisciplinary stabilization. This case report has been prepared in accordance with the SCARE 2025 guidelines^[^7^]^.

## Case presentation

We present the case of a preterm female neonate born via spontaneous vaginal delivery to a 27-year-old mother, gravida 2, with a history of one prior abortion. The pregnancy was complicated by gestational diabetes mellitus, for which the mother was receiving metformin 500 mg three times daily. There were no other reported maternal comorbidities. The parents were non-consanguineous and had no history of tobacco use. The mother reported a vaginal infection during the first trimester. Antenatal ultrasonography performed during the first and early second trimesters was unremarkable. However, a detailed anomaly scan at 28 weeks’ gestation demonstrated abnormal cranial findings suggestive of semilobar HPE. No other congenital anomalies were identified antenatally.

**Figure 1.**
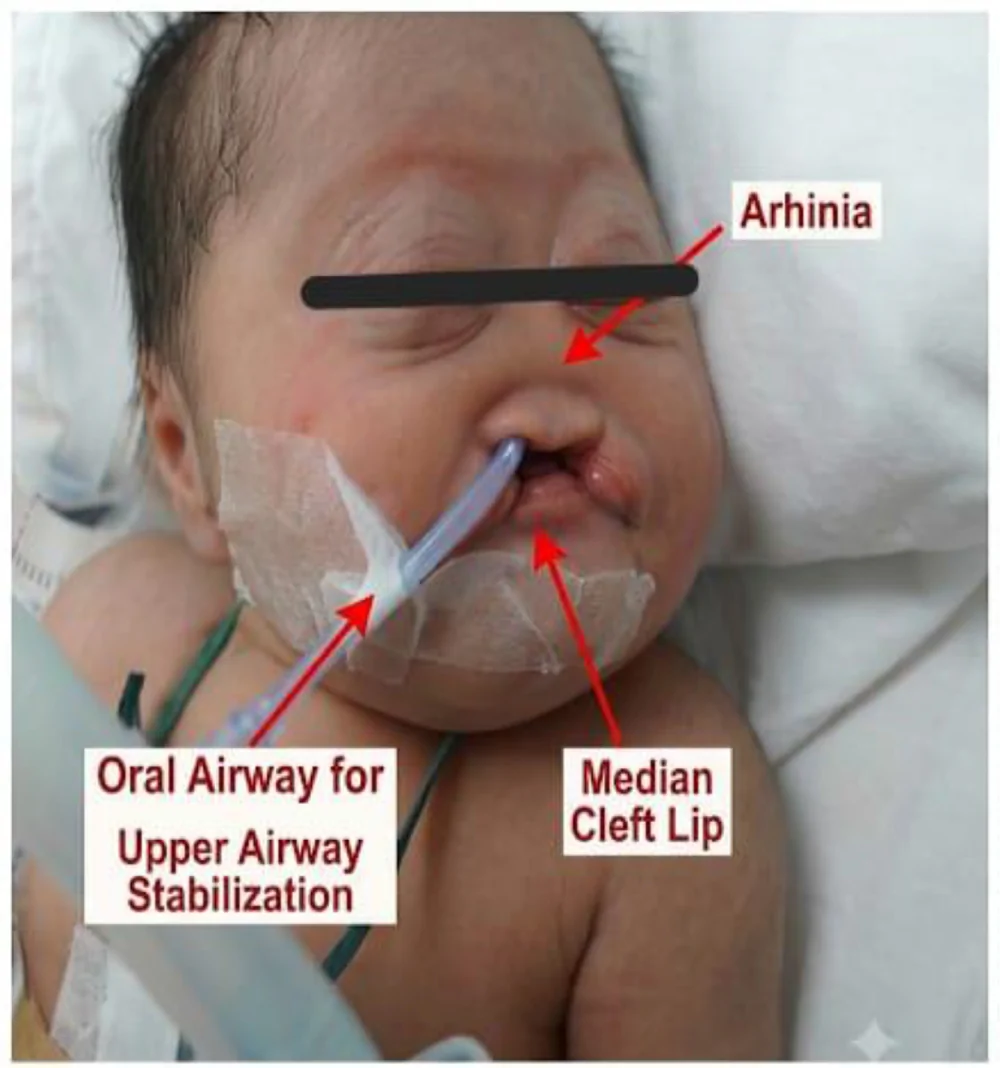
Neonatal facial phenotype at birth. A clinical photograph shows profound midline dysmorphism, including microcephaly, total arhinia with a smooth midfacial surface, a median cleft lip and palate, and low-set ears.

The neonate was delivered preterm and weighed 1900 g at birth. She cried on stimulation, with Apgar scores of 6/10 at 1 minute, 8/10 at 5 minutes, and 10/10 at 10 minutes. On initial assessment, the newborn was hemodynamically stable, with a heart rate of 111 beats per minute, a respiratory rate of 62 breaths per minute, a temperature of 37°C, and oxygen saturation maintained on 1 L/min of supplemental oxygen. Physical examination at birth revealed profound midline dysmorphism. Most notably, the infant exhibited total arhinia, characterized by a smooth, flat midfacial surface with a complete absence of the external nose, nasal bridge, and nares. This was accompanied by microcephaly, median cleft lip and palate, low-set ears, premaxillary agenesis, and swollen eyelids (Fig. 1). Neurological examination showed generalized hypotonia, poor sucking, and absence of primitive reflexes. Cardiovascular and respiratory examinations were unremarkable, and the external genitalia were normal. Supplemental oxygen was discontinued by the second postnatal day as the infant successfully adapted to reflexive mouth breathing.

**Figure 2.**
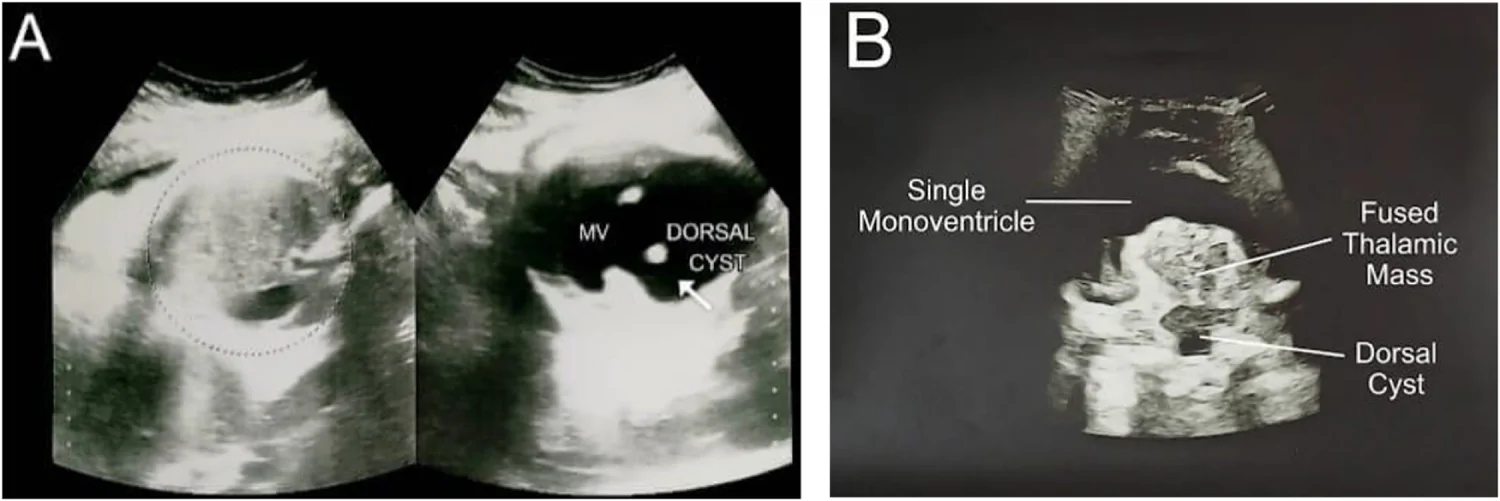
Antenatal and postnatal ultrasonography. (A) Antenatal anomaly scan at 28 weeks’ gestation demonstrating abnormal cranial architecture suggestive of semilobar holoprosencephaly. (B) Postnatal cranial ultrasound confirming a large single midline monoventricle, fused thalami, and a partially formed interhemispheric fissure.

Postnatal cranial ultrasonography demonstrated findings consistent with semilobar HPE, including a large single midline monoventricle, fused thalami, a partially formed interhemispheric fissure, the presence of the falx cerebri anteriorly with posterior deficiency, and the absence of the septum pellucidum (Fig. 2). These findings were consistent with the abnormalities identified on the antenatal scan performed at 28 weeks’ gestation. Abdominopelvic ultrasonography revealed an intact abdominal wall, a normal-appearing liver, bilateral kidneys, and intestinal loops with normal echogenicity. Echocardiography showed no structural cardiac anomalies.

Based on these findings, several differential diagnoses were systematically evaluated. Alobar HPE was ruled out by the presence of a partially formed posterior interhemispheric fissure, while lobar variants were excluded due to extensive anterior thalamic fusion, solidifying a semilobar configuration. Isolated congenital arhinia was discounted as it is characterized by intact intracranial architecture, which directly contradicts our patient’s profound cortical fusion. Frontonasal dysplasia was also excluded, as it typically presents with hypertelorism and a bifid nose rather than total arhinia. Furthermore, the absence of postaxial polydactyly, microphthalmia, or visceral and cardiac defects on systemic screening strongly argued against chromosomal trisomies, such as Patau syndrome (trisomy 13). While a definitive genetic etiology could not be confirmed because next-generation sequencing (NGS) and chromosomal microarray testing were unavailable in our resource-limited setting and antenatal care was limited, the clinical picture remains highly distinct. Synthesizing the anterior ventricular fusion, total arhinia, median cleft lip and palate, and maternal history of unmanaged gestational diabetes, the team established a provisional diagnosis of non-syndromic semilobar HPE with complete congenital arhinia and median cleft lip and palate, secondary to maternal diabetic teratogenesis.

**Figure 3.**
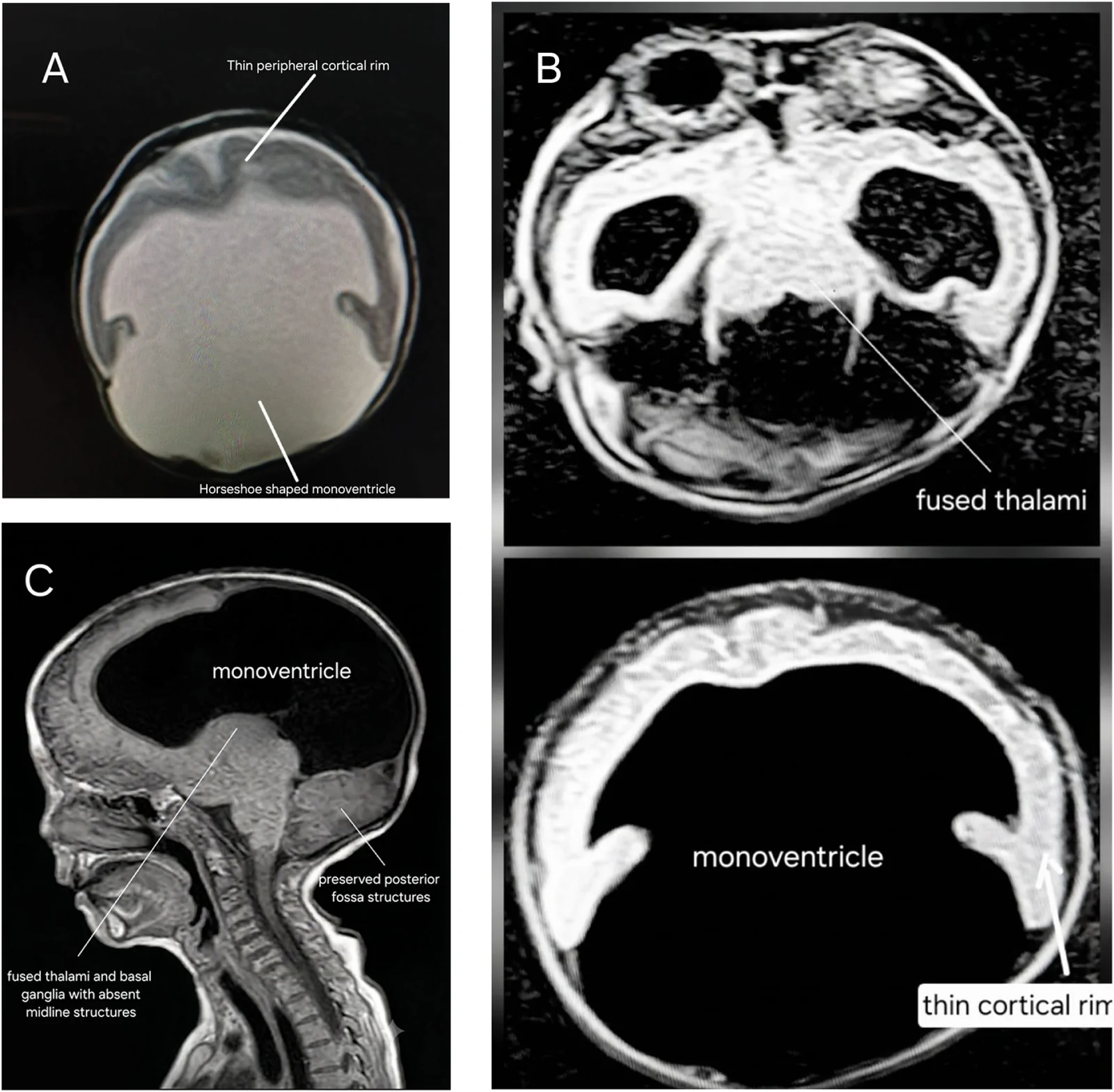
Brain MRI axial T2W (A) and axial FLAIR (B) sequences showing a large, horseshoe-shaped monoventricle with a thin peripheral cortical rim and complete suppression of CSF signal on FLAIR. The sagittal (C) image demonstrates fused thalami and basal ganglia at the midline with absence of the septum pellucidum and anterior falx cerebri, characteristic of the semilobar variant.

**Figure 4.**
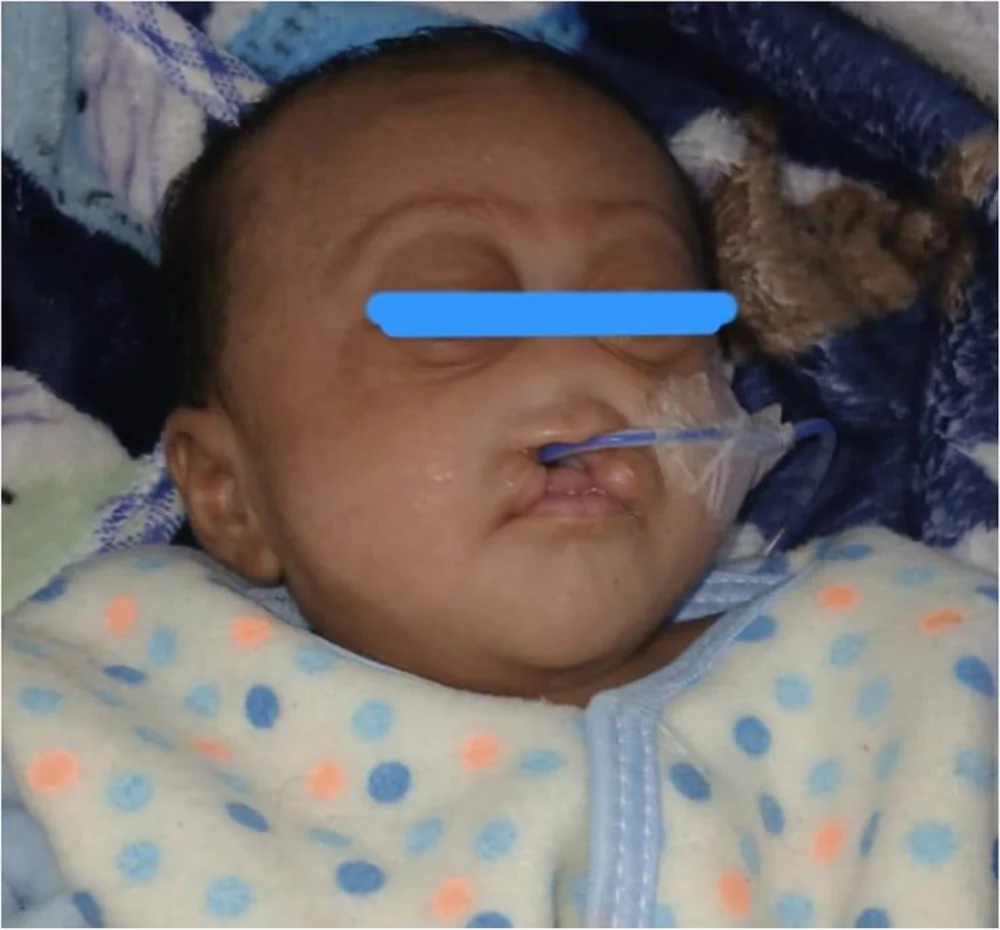
An infant at 1 month of age demonstrating the necessity for persistent orogastric (OG) tube feeding to manage feeding-breathing discoordination caused by total arhinia.

The neonate received supportive care in the neonatal unit and was discharged with detailed parental counseling regarding the guarded prognosis and the need for specialized care. At one month of age, the infant was readmitted with high-grade fever and multiple seizure episodes. Magnetic resonance imaging of the brain revealed features consistent with semilobar HPE, including an enlarged, horseshoe-shaped monoventricular cavity surrounded by a thin rim of cerebral cortex; absence of the falx cerebri; fused thalami; and a normal cerebellum and brainstem. The internal nasal architecture, including the septum and turbinates, was absent, correlating with the clinical diagnosis of arhinia (Fig. 3). No additional organic pathology was identified. Cerebrospinal fluid analysis following lumbar puncture showed elevated protein levels with normal glucose and lactate dehydrogenase values. Cytological examination was negative for malignant cells. The infant was treated symptomatically for fever and seizures and, after clinical stabilization, was discharged on antiepileptic medication, with advice for close follow-up. Due to the arhinia, nutritional support was continued via an OG tube (Fig. 4).

Following this initial stabilization, the patient was transitioned to a structured, home-based pediatric palliative care model. Over the subsequent 8 months of longitudinal follow-up, the infant demonstrated remarkable clinical stabilization. Notably, her initial epilepsy was successfully controlled by antiepileptic therapy, and she has uniquely remained free from recurrent clinical seizures, suggesting that pharmacological stabilization of the cortical threshold was pivotal in preventing further neuro-excitability. However, her clinical course remains challenged by underlying physiological vulnerability, underscored by a persistent dependence on OG tube feeding due to her severe oromotor limitations. This fragility was further evidenced by a recent acute readmission for hyperpyrexia secondary to a laboratory-confirmed urinary tract infection (UTI), highlighting that while catastrophic primary cortical events have been mitigated, the patient remains highly susceptible to systemic infections and secondary metabolic dysregulation.

## Discussion

The pathogenesis of HPE in this neonate provides a compelling illustration of the catastrophic consequences that occur when the primary inductive signals of the ventral forebrain are disrupted during early gastrulation^[^1,2^]^. The emergence of semilobar HPE signifies a partial success in the cleavage process, in which the posterior prosencephalon manages to initiate hemispheric separation while the anterior structures remain inextricably fused. This case is particularly noteworthy because the presence of total arhinia and premaxillary agenesis typically heralds the more primitive alobar form, yet the radiological evidence of a partially formed interhemispheric fissure and a posterior falx cerebri confirms a higher level of neuroanatomical organization than the facial phenotype suggests. Glista F. *et al* demonstrated that individuals carrying the same SHH mutation could present with vastly different phenotypes, ranging from severe alobar HPE to “microforms” such as a single central maxillary incisor, hypotelorism, or even a completely normal clinical appearance^[^8^]^. This structural discrepancy rules out simple, isolated congenital arhinia or frontonasal dysplasia, where intracranial cleavage remains intact, and instead underscores a non-linear relationship between facial morphogenesis and cerebral cleavage. This suggests that the signaling thresholds required for nasal placode development may be more sensitive to environmental or metabolic insults than those governing the posterior expansion of the telencephalon.

A critical diagnostic consideration in any case of HPE is the association with Patau syndrome (Trisomy 13), the most frequent chromosomal abnormality associated with this spectrum. The literature suggests that approximately 24–45% of infants with Patau syndrome exhibit some form of HPE, and conversely, up to 75% of HPE cases with an underlying cytogenetic cause are attributed to Trisomy 13^[^9^]^. While the “cardinal triad” of Patau syndrome consisting of orofacial clefts, microphthalmia, and postaxial polydactyly was only partially present in our patient via evidenced by her severe median cleft lip and palate, the intensity of the craniofacial dysmorphism and the early-onset seizures strongly mimic this syndromic phenotype. In this instance, the absence of structural cardiac or renal anomalies on ultrasonography and the lack of polydactyly point toward a non-syndromic or environmental presentation driven by maternal diabetes. Advanced cytogenetic testing, such as chromosomal microarray analysis or NGS, was unavailable in our resource-limited setting, which constituted the primary diagnostic limitation of this report. Consequently, a definitive genetic exclusion remains impossible, and the distinction between syndromic and non-syndromic HPE had to be inferred solely from the constellation of clinical and radiological findings^[^10^]^. This challenge reflects a broader global clinical reality where complex neurodevelopmental anomalies must often be managed without the aid of molecular confirmation. HPE is characterized by a highly complex, heterogeneous genetic architecture, often driven by pathogenic variants in master regulatory genes such as SHH, ZIC2, SIX3, and TGIF1. Without molecular sequencing, it is impossible to definitively delineate the relative contributions of submicroscopic genetic anomalies versus the environmental metabolic influence of maternal gestational diabetes. Nevertheless, it is biologically plausible that an unmapped, low-penetrance genetic variant or a de novo mutation acted synergistically with the altered maternal metabolic state to precipitate this severe phenotypic discordance^[^11^]^.

The potential role of maternal gestational diabetes and concurrent metformin therapy adds a further layer of metabolic complexity to the etiologic discussion. Among documented teratogenic exposures, maternal hyperglycemia remains an established risk factor for HPE spectrum disorders, with historical epidemiological data indicating a significantly elevated risk in infants born to diabetic mothers compared with non-diabetic controls^[^12,13^]^. While the association between pre-gestational diabetes and neural tube defects is well characterized, the exact pathogenetic link between managed gestational diabetes and intermediate HPE variants remains an area of active clinical investigation. From an embryological standpoint, it is biologically plausible that transient maternal hyperinsulinemia or hyperglycemia may serve as a metabolic antagonist to embryonic cholesterol biosynthesis and downstream SHH signaling during critical windows of midline patterning^[^14^]^. Because the SHH protein requires covalent cholesterol modification to function correctly, this metabolic interference is hypothesized to have acted as an environmental disruptor, potentially explaining why the highly sensitive nasal placode development was pushed toward severe arhinia despite a relatively more preserved posterior cerebral architecture. However, because these observations are drawn from an isolated case report, a direct causal relationship between maternal gestational diabetes and this specific phenotypic presentation cannot be definitively concluded. This pathogenetic pathway is presented strictly as an intellectually interesting hypothesis that requires further molecular study.

When compared to recent clinical literature, our patient’s presentation provides a unique contribution to the evolving understanding of the HPE spectrum. While the exceptional coexistence of semilobar HPE and arhinia has been recently documented in liveborn infants by Kebbeh and Nawfal^[^15^]^, our report extends this narrative by documenting longitudinal survival, a departure from the early mortality historically associated with this rare anatomical pairing. Furthermore, while HPE pathogenesis is frequently investigated via genetic sequencing (e.g., ZIC2 variant analysis as described by Nonkulovski and Sofijanova^[^16^]^), our case emphasizes the significant, potentially independent role of environmental metabolic stressors. Although the lack of molecular testing precludes definitive exclusion of a causative genetic variant, our patient’s clinical profile lacks the classical syndromic dysmorphism often reported in genetically confirmed HPE cohorts. Instead, the history of maternal gestational diabetes provides a clinically significant environmental context; given that maternal hyperglycemia is a recognized disruptor of cholesterol-dependent SHH signaling, this metabolic stressor likely served as a plausible contributor to the patient’s developmental trajectory. This is further corroborated by recent evidence from Çelik *et al*^[^17^]^, reinforcing a potential link between glycemic dysregulation and midline developmental arrest. Most significantly, our patient’s sustained stabilization mirrors recent clinical evidence demonstrating that extended survival is entirely achievable within this spectrum. This aligns with the findings of Pallangyo *et al*^[^18^]^, who documented survival up to 12 months in a child with semilobar HPE linked to maternal diabetes, as well as Gabr^[^19^]^, who demonstrated that tailored supportive protocols can facilitate unexpected longitudinal survival even in the most severe alobar phenotypes. Collectively, these studies underscore that with rigorous multidisciplinary, home-based care focusing on caloric titration and autonomic management long-term systemic stability is achievable, even in cases of profound structural vulnerability, regardless of the underlying molecular etiology.

A notable feature of this patient’s clinical course is her sustained systemic stability: despite the severe midline disruption and the metabolic insult associated with maternal diabetes, she has remained free from acute systemic morbidity throughout her 8-month follow-up. This favorable trajectory is attributed to a proactive, multidisciplinary care model focused on stabilizing the patient’s precarious equilibrium through rigorous caloric titration, preemptive screening for infectious triggers of hypothalamic instability, and consistent caregiver-led monitoring of autonomic indicators^[^1,2^]^. By mitigating secondary risks such as aspiration-related pneumonia and electrolyte dysregulation, this approach has prevented the progressive systemic deterioration often associated with severe HPE. However, the patient’s ongoing clinical trajectory continues to underscore the chronic physiological fragility inherent in these malformations. While the patient’s recurrent episodes of hyperpyrexia are clinically consistent with hypothalamic thermoregulatory dysfunction, this diagnostic hypothesis must be treated with appropriate academic caution^[^1^]^. In the absence of continuous physiological monitoring, formal neuroendocrine profiling, or histopathological evidence, the differentiation between primary central diencephalic fever and secondary systemic responses to peripheral triggers remains presumptive. It is clinically plausible that the patient’s structural anomalies confer a lowered threshold for temperature instability, causing central regulatory mechanisms to respond disproportionately to minor systemic stressors, such as the recently documented UTI^[^20^]^. Furthermore, the persistent necessity for OG tube feeding reflects the mechanical constraints imposed by arhinia, where the oral cavity is monopolized by the requirements of reflexive breathing^[^5,15^]^. Looking forward, any surgical management of the patient’s cleft lip and palate, as well as arhinia, must be approached with academic caution; a staged, functional-first strategy will be essential, prioritizing airway patency and nutritional safety over esthetic reconstruction. Ultimately, while the patient’s long-term outlook remains guarded and contingent on monitoring for delayed-onset hydrocephalus and endocrine insufficiency, her survival demonstrates that a proactive, home-based, and multidisciplinary management model can successfully extend life beyond the neonatal period, even in the presence of persistent autonomic and regulatory vulnerabilities^[^18,19^]^.

## Conclusion

This case highlights a rare phenotypic discordance in which severe anterior facial dysmorphism occurs alongside partially preserved cerebral cleavage in semilobar HPE. While the etiology is likely multifactorial, involving the potentially synergistic effects of maternal hyperglycemia and genetic susceptibility, the clinical significance lies in the patient’s unexpected long-term survival. This report demonstrates that even in the presence of profound structural and autonomic vulnerabilities, proactive, multidisciplinary management can effectively mitigate secondary systemic morbidity and support survival beyond the neonatal period. This model provides a functional framework for stabilizing patients with severe HPE through consistent, home-based surveillance and multidisciplinary support.

## Data Availability

Data sharing is not applicable as no datasets were generated.
